# Supplementary figures and images for: Assessment of potential transthyretin amyloid cardiomyopathy cases in the Brazilian public health system using a machine learning model
Source: PLoS One. 2024 Feb 15;19(2):e0278738. doi: 10.1371/journal.pone.0278738 (PMC10868784; doi:10.1371/journal.pone.0278738)

SUPPLEMENTARY MATERIAL

S1 Figure. Supervised machine learning model approach


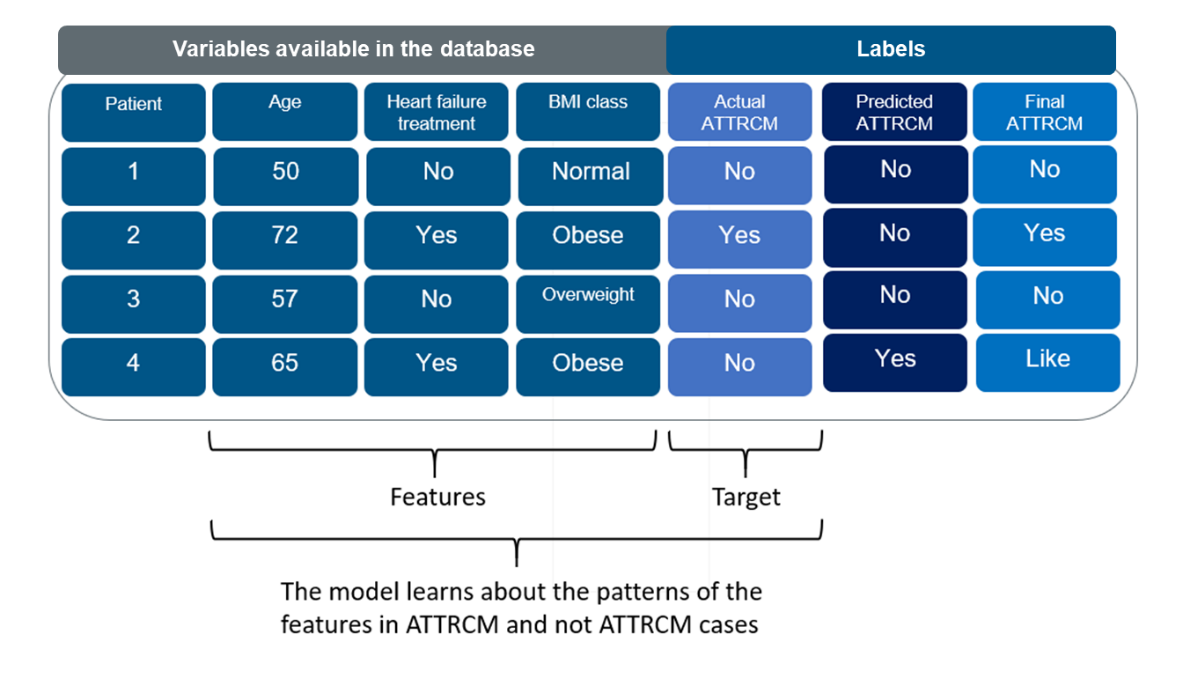

Supplement: S1 Fig — (DOCX) [file pone.0278738.s001.docx]

S2 Figure. DATASUS databases description


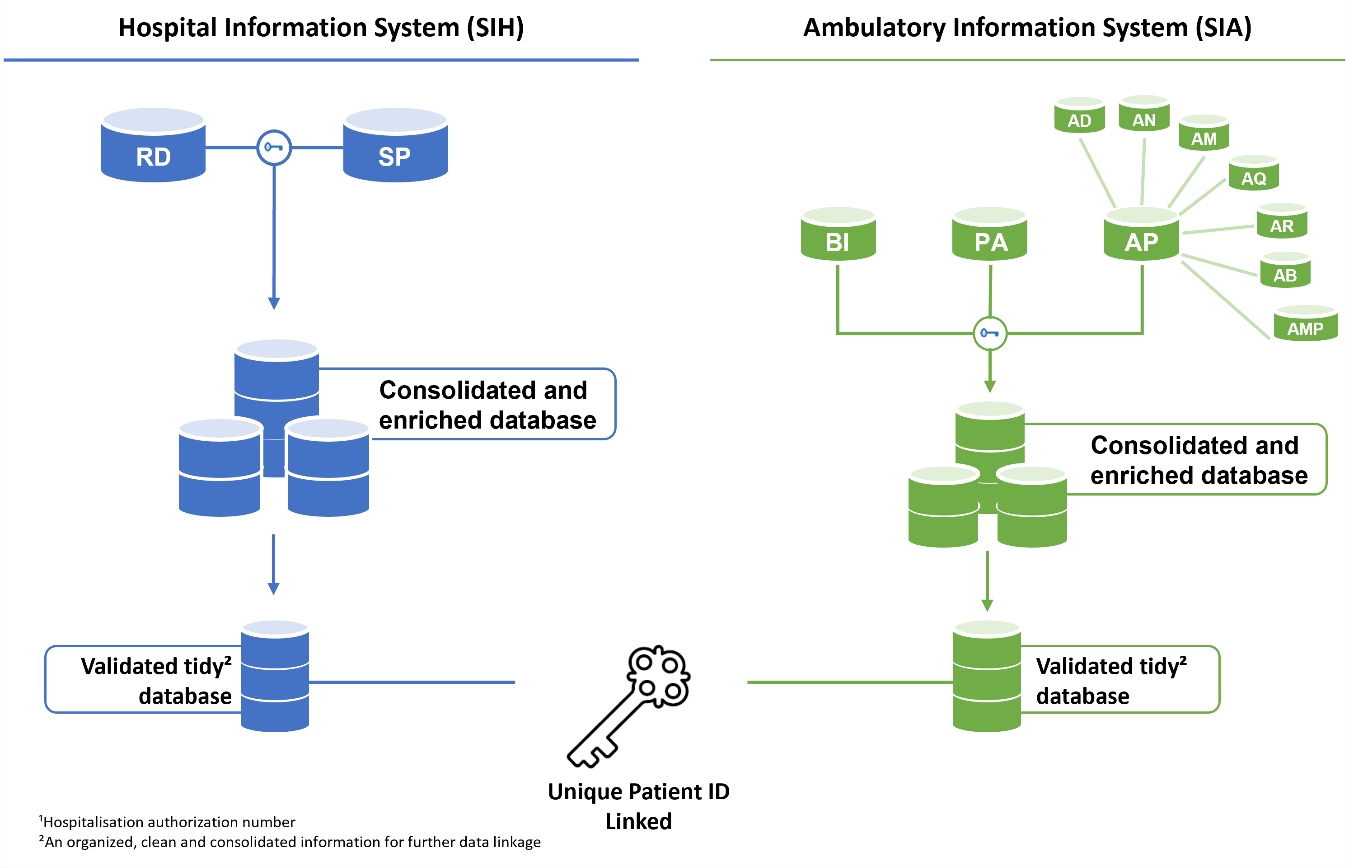

Supplement: S2 Fig — (DOCX) [file pone.0278738.s002.docx]

S3 Figure. Machine learning model approach


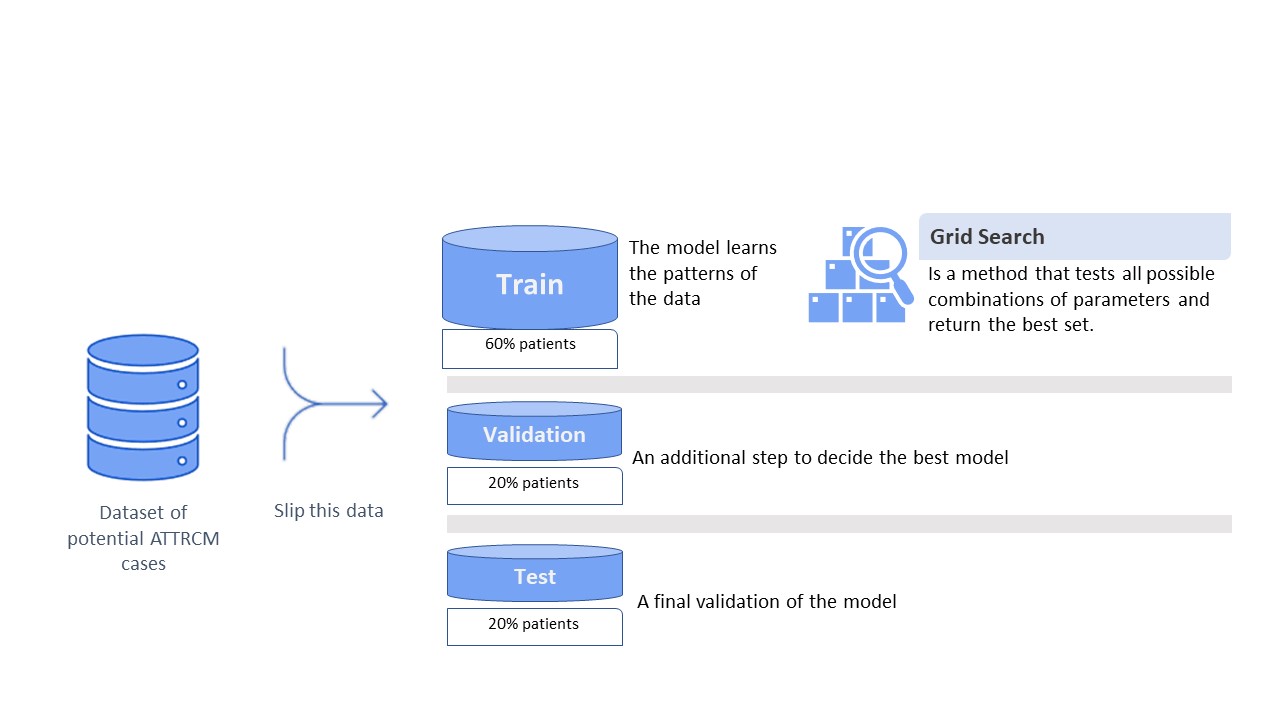

Supplement: S3 Fig — (DOCX) [file pone.0278738.s003.docx]
