# Supplementary material for: Assessment of potential transthyretin amyloid cardiomyopathy cases in the Brazilian public health system using a machine learning model
Source: PLoS One. 2024 Feb 15;19(2):e0278738. doi: 10.1371/journal.pone.0278738 (PMC10868784; doi:10.1371/journal.pone.0278738)
Supplement: S4 Fig — (DOCX) [file pone.0278738.s004.docx]

S4 Figure. Equations for Accuracy, Sensitivity and Specificity calculation


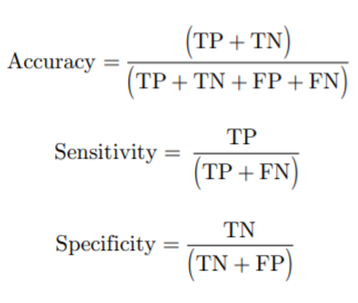


TP: true positive; TN: true negative; FP: false positive; FN: false negative
