# Supplementary material for: Assessment of potential transthyretin amyloid cardiomyopathy cases in the Brazilian public health system using a machine learning model
Source: PLoS One. 2024 Feb 15;19(2):e0278738. doi: 10.1371/journal.pone.0278738 (PMC10868784; doi:10.1371/journal.pone.0278738)
Supplement: S1 Table — (DOCX) [file pone.0278738.s005.docx]

S1 Table. Mandatory and secondary procedures considered for classifying ATTR-CM and ATTR-CM-like cases

| **Mandatory procedures** |
| --- |
| Heart transplantation |
| Heart biopsy |
| Tafamidis 20 mg* |
| Bone scintigraphy of joints and/or bones |
| Heart magnetic resonance |
| **Secondary procedures** |
| Treatment of hypertrophic heart disease |
| Treatment of myocardiopathies |
| Treatment of arrhythmias |
| Heart failure treatment |
| Surgical treatment of carpal tunnel syndrome |
| Tenosynovectomy in upper limb |
| Electrocardiogram |
| Transthoracic echocardiography |
| Transesophageal echocardiography |
| Skin and soft parts biopsy |
| Troponin dosage |
| Dosage BNP and NT-proBNP |
| Endocavitary cardiac pacemaker implant |
| Epimyocardial heart pacer implant |
| Transvenous heart pacemaker implant |
| Epimyocardial dual chamber pacemaker implant |
| Transvenous dual chamber pacemaker implant |
| Epimyocardial single chamber pacemaker implant |
| Transvenous single chamber pacemaker implant |
| Valve prosthesis implant |
| *Tafamidis was one of the selected features used in the model to identify patients with cardiac manifestation. For example: if a patient had a claim with ICD-10 I50.0 (congestive heart failure) and a record of tafamidis use, it is possible that he has ATTR amyloidosis with cardiac manifestation. The use of tafamidis was only considered to identify wild-type ATTR patients, since hereditary ATTR might use tafamidis even if the patient only has polyneuropathy. Also, the model classified the cases considering not only the use of tafamidis, but also the ICD-10 codes selected, and other variables considered for the study. |
