# Supplementary material for: Assessment of potential transthyretin amyloid cardiomyopathy cases in the Brazilian public health system using a machine learning model
Source: PLoS One. 2024 Feb 15;19(2):e0278738. doi: 10.1371/journal.pone.0278738 (PMC10868784; doi:10.1371/journal.pone.0278738)
Supplement: S3 Table — (DOCX) [file pone.0278738.s007.docx]

S4 Table. Top 20 procedures for wtATTR-CM patients (number of unique patients)

|  | **Procedures** | **wtATTR-CM** | | |
| --- | --- | --- | --- | --- |
|  |  | **Total** | **wtATTR-CM reference** | **wtATTR-CM-like** |
|  |  | 1581 | 203 | 1378 |
| 1 | 0303060212 HEART FAILURE TREATMENT | 1483 (93.8%) | 168 (82.76%) | 1315 (95.43%) |
| 2 | 0303030046 METABOLIC DISEASE TREATMENT | 85 (5.38%) | 21 (10.34%) | 64 (4.64%) |
| 3 | 0205010032 TRANSTHORACIC ECHOCARDIOGRAM | 76 (4.81%) | 13 (6.4%) | 63 (4.57%) |
| 4 | 0203020030 ANATOMO-PATHOLOGICAL EXAMINATION BIOPSY | 81 (5.12%) | 22 (10.84%) | 59 (4.28%) |
| 5 | 0301010072 MEDICAL APPOINTMENT IN SPECIALIZED CARE | 132 (8.35%) | 24 (11.82%) | 108 (7.84%) |
| 6 | 0305020056 CHRONIC KIDNEY FAILURE TREATMENT | 264 (16.7%) | 47 (23.15%) | 217 (15.75%) |
| 7 | 0205020046 TOTAL ABDOMEN ULTRASOUND | 1 (0.06%) | 1 (0.49%) | 0 (0%) |
| 8 | 0303060026 TREATMENT OF ARRYTHMIAS | 126 (7.97%) | 4 (1.97%) | 122 (8.85%) |
| 9 | 0202030679 ASSESSMENT OF ANTI HEPATITIS C VIRUS ANTIBODIES | 250 (15.81%) | 45 (22.17%) | 205 (14.88%) |
| 10 | 0211060259 TONOMETRY | 107 (6.77%) | 12 (5.91%) | 95 (6.89%) |
| 11 | 0406010650 HEART PACEMAKER IMPLANTATION | 66 (4.17%) | 9 (4.43%) | 57 (4.14%) |
| 12 | 0202031209 TROPONIN DOSAGE | 71 (4.49%) | 16 (7.88%) | 55 (3.99%) |
| 13 | 0205010040 DOPPLER ULTRASOUND | 225 (14.23%) | 23 (11.33%) | 202 (14.66%) |
| 14 | 0206020031 TOMOGRAPHY | 103 (6.51%) | 102 (50.25%) | 1 (0.07%) |
| 15 | 0211060127 RETINA MAPPING | 806 (50.98%) | 73 (35.96%) | 733 (53.19%) |
| 16 | 0205020054 URINARY SYSTEM ULTRASOUND | 240 (15.18%) | 36 (17.73%) | 204 (14.8%) |
| 17 | 0202020380 COMPLETE HEMOGRAM | 253 (16%) | 65 (32.02%) | 188 (13.64%) |
| 18 | 0202010694 UREA DOSAGE | 27 (1.71%) | 6 (2.96%) | 21 (1.52%) |
| 19 | 0302050027 PHYSIOTHERAPY CARE IN MOTOR CHANGES | 108 (6.83%) | 14 (6.9%) | 94 (6.82%) |
| 20 | 0202010600 POTASSIUM DOSAGE | 167 (10.56%) | 25 (12.32%) | 142 (10.3%) |
| * Only procedures claim with the selected ATTRCM-related or cardiac-related ICD-10 codes | | |  |  |
