# Supplementary material for: Assessment of potential transthyretin amyloid cardiomyopathy cases in the Brazilian public health system using a machine learning model
Source: PLoS One. 2024 Feb 15;19(2):e0278738. doi: 10.1371/journal.pone.0278738 (PMC10868784; doi:10.1371/journal.pone.0278738)
Supplement: Attachment — Submitted filename: peer review.pdf [file pone.0278738.s009.pdf]

Comments for authors:
The authors use machine learning models to identify and classify ATTR-CM, an amyloid fibril that can cause cardiac arrest by preventing the ventricles from pumping blood effectively to the rest of the body. The study was conducted using patient data collected between 2015 and 2021 in order to assist the Brazilian health care system in enhancing patient health guidelines.

-In the introduction I suggest to authors to read and refer to this article which explains in general what amyloid fibrils are: Auriemma Citarella, Alessia, et al. "ENTAIL: yEt aNoTher amyloid fIbrils cLassifier." BMC bioinformatics 23.1 (2022): 1-15.
-In the introduction I suggest putting a short introduction to cardiovascular disease in general and referring to these papers:
De Marco, Fabiola, et al. "Classification of QRS complexes to detect Premature Ventricular Contraction using machine learning techniques." Plos one 17.8 (2022): e0268555.
De Marco, Fabiola, Dewar Finlay, and Raymond R. Bond. "Classification of Premature Ventricular Contraction Using Deep Learning." 2020 Computing in Cardiology. IEEE, 2020.
-At the end of the introduction, I suggest inserting a brief description of the objective of the paper, the dataset used and a very brief description of the results achieved.
- In the introduction, add structure to the paper to make it easier to read.

-Check the references in the method section.
-What are the fields of the dataset? How many people are involved in the study? Clarify and include a better description of the dataset.
-A misunderstanding exists between dataset parameters and model input parameters. Are they identical? Are only some selected? Then why?
-Why are so many more samples taken from people over 50? Does this occur more frequently in people over 50? Explain this point.
-Insert a diagram or table to explain the structure of the dataset well, explaining what they are for each field and category in order to favor the reader.
-What percentage are training, validation and testing chosen?
-Why wasn't the AUC metric used? please if it is possible to insert it.

-The description part of the dataset in the results section should be moved to the dataset subsection.
-It is unclear what descriptions images and tables refer to in the results section. Need to double check and fix. In this way I cannot say that they are clear and adequate

-The conclusion needs improvement. In this way, they cannot be accepted. The authors should provide a summary of their work, report a portion of the findings, and explain how their work has improved the domain.

Comments for editor:
I recommend a major revision of this paper due to the fact that their research has the potential to improve the prevention and prognosis of this disease, but must be revised and improved to be accepted.
