# Supplementary material for: Assessment of potential transthyretin amyloid cardiomyopathy cases in the Brazilian public health system using a machine learning model
Source: PLoS One. 2024 Feb 15;19(2):e0278738. doi: 10.1371/journal.pone.0278738 (PMC10868784; doi:10.1371/journal.pone.0278738)
Supplement: Attachment — Submitted filename: revision.docx [file pone.0278738.s010.docx]

Thank you for the opportunity to review this paper from Laper et colleagues.

I really appreciate the effort to estimate the prevalence of cardiac amyloidosis in a wide national population and I think that machine learning models represent the future of screening for cardiomyopathies and the subject is of great interest, however I have to report my concern about the accuracy of the data presented.

Major revisions:

Labelling data as “reference ATTR-CM” and “not ATTR-CM” was defined by the investigators, potentially introducing a bias, specifically I do not agree with the definitions of “ATTR-CM-like”. From my understanding, in methods you stated that for diagnosis of “ATTR-CM-like” you need 1 cardiac related ICD-10 AND 1 secondary procedure AND 1 mandatory procedure. let me give you an example fulfilling your criteria: patient with a cardiac related ICD 10 AND heart magnetic resonance (mandatory) AND troponin dosage… this example for me it is not suggestive of ATTR-CM unless CMR presents features consistent with amyloid cardiomyopathy, but this is not specified in the text or at least for me it is unclear. I can provide you plenty of combinations that you labelled as “ATTR-CM-like” but they are hardly acceptable.

Similarly with the second possible definition of “ATTR-CM-like” e.g. ICD 10-related wtATTR (organ limited amyloidosis) AND secondary procedure (ECG), it is difficult to accept that this combination is suggestive of “ATTR-CM-like”. For what we know it could be amyloidosis in the carpal tunnel or just in the lung tissue or many other different alternatives but not necessarily “ATTR-CM-like”, isn’t it?

My concern is similar for “hATTR-CM -like”, I provide you another example fulfilling your criteria for “hATTR-CM -like”: Neuropathic heredofamilial amyloidosis (ICD10 criteria) AND skin biopsy (secondary criteria), for me it does not represent a “hATTR-CM-like” but it could simply be a neuropathic amyloidosis with skin biopsy performed and no cardiac involvement.

Secondary procedures criteria may often be just screening procedure? The fact that a patient performed a cardiological screening does not mean that it resulted positive to it.

What I trying to prove is that the criteria you have chosen are non specific for amyloid cardiac involvement, or at least are questionable.

I think that we have internationally shared specific criteria for ATTR cardiac involvement, I can accept the fact that in such a large population, referring to ATTR related IC10 can be a valid option however ATTR related ICD-10 are non specific for cardiac involvement and the association with “cardiac related ICD 10” can somehow increase the specificity but the overall result might be unprecise either in “ATTR-CM- like” or in “ATTR-CM-reference” (e.g. neuropathic heredofamilial amyloidosis + aortic valve stenosis fulfill you “ATTR-CM-reference” criteria but for me it can just be a neuropathic amyloidosis with a degree of aortic stenosis in an old patient without amyloidosis proven cardiac involvement)

In conclusion I appreciate the effort but I have to express my concern on most of the definitions you provided and consequently to all the analysis derived from them.

You excluded patients with “blood cancer” to avoid overlap with AL however, all patients with a positive serum or urinary immunofixation rise the suspect of possible AL ( independently from the presence of a blood cancer) and only biopsy proven ATTR should be included, I don’t think this has been done in your study , rising additional biases

Minor revisions:

Is it normal that in the manuscript I repeatedly found “(Supplementary **Error! Reference source not found.**)”? Maybe there is something wrong in my downloaded manuscript, but it is difficult to understand the refences in the text.

For me it is unclear how you tested the accuracy, sensitivity and specificity of the ML model, from what I understood you classified wtATTR-CM and hATTR-CM based on ICD-10 with criteria not accepted internationally for the diagnosis of cardiac amyloidosis.
