# Supplementary material for: Assessment of potential transthyretin amyloid cardiomyopathy cases in the Brazilian public health system using a machine learning model
Source: PLoS One. 2024 Feb 15;19(2):e0278738. doi: 10.1371/journal.pone.0278738 (PMC10868784; doi:10.1371/journal.pone.0278738)
Supplement: Attachment — Submitted filename: Response to Reviewers_r1_v_submitted.docx [file pone.0278738.s011.docx]

**Reviewer 1**

1. **Labelling data as “reference ATTR-CM” and “not ATTR-CM” was defined by the investigators, potentially introducing a bias, specifically I do not agree with the definitions of “ATTR-CM-like”. From my understanding, in methods you stated that for diagnosis of “ATTR-CM-like” you need 1 cardiac related ICD-10 AND 1 secondary procedure AND 1 mandatory procedure. let me give you an example fulfilling your criteria: patient with a cardiac related ICD 10 AND heart magnetic resonance (mandatory) AND troponin dosage… this example for me it is not suggestive of ATTR-CM unless CMR presents features consistent with amyloid cardiomyopathy, but this is not specified in the text or at least for me it is unclear. I can provide you plenty of combinations that you labelled as “ATTR-CM-like” but they are hardly acceptable.**

***Response:*** *Thank you for your comment. We have specified in the text clearly the model steps for classification and data source characteristics, as well as the use of an expert panel to mitigate such points mentioned.*

*Now the text reflects clearly that those patients were included as potential ATTR-CM cases and then classified with the machine learning model as ATTR-CM-like (lines 95 to 180). This was assessed by the expert panel to identify and propose mitigations on the classification label construction, as you kindly appointed since the dataset did not provide enough clinical information for a deeper assessment. We also stated this better in the study limitations (lines 548 to 569).*

2. **Similarly with the second possible definition of “ATTR-CM-like” e.g., ICD 10-related wtATTR (organ limited amyloidosis) AND secondary procedure (ECG), it is difficult to accept that this combination is suggestive of “ATTR-CM-like”. For what we know it could be amyloidosis in the carpal tunnel or just in the lung tissue or many other different alternatives but not necessarily “ATTR-CM-like”, isn’t it?**

***Response:*** *Thank you for your comment. As mentioned in the answer to your previous comment, we have clarified the methodology for the model label eligibility criteria construction and expert panel validation. We also reflected this in the study limitations.*

3. **My concern is similar for “hATTR-CM -like”, I provide you another example fulfilling your criteria for “hATTR-CM -like”: Neuropathic heredofamilial amyloidosis (ICD10 criteria) AND skin biopsy (secondary criteria), for me it does not represent a “hATTR-CM-like” but it could simply be a neuropathic amyloidosis with skin biopsy performed and no cardiac involvement.**

***Response:*** *Thank you for your comment. As mentioned in the answer to your previous comment 1, we have clarified the methodology for the model label eligibility criteria construction and expert panel validation. We also reflected this in the study limitations.*

4. **Secondary procedures criteria may often be just screening procedure? The fact that a patient performed a cardiological screening does not mean that it resulted positive to it.**

***Response:*** *Thank you for your comment. We clarified in the methodology that the inclusion criteria were to define the population to have the machine learning model applied (lines 95 to 180). Besides that, it is important to highlight that as this is an administrative claim database study, we have some limitations regarding lab results. Those were also stated in the study limitations text (lines (564 to 569).*

**What I trying to prove is that the criteria you have chosen are nonspecific for amyloid cardiac involvement, or at least are questionable.**

**I think that we have internationally shared specific criteria for ATTR cardiac involvement, I can accept the fact that in such a large population, referring to ATTR related IC10 can be a valid option, however, ATTR related ICD-10 are non specific for cardiac involvement and the association with “cardiac related ICD 10” can somehow increase the specificity but the overall result might be unprecise either in “ATTR-CM- like” or in “ATTR-CM-reference” (e.g. neuropathic heredofamilial amyloidosis + aortic valve stenosis fulfill you “ATTR-CM-reference” criteria but for me it can just be a neuropathic amyloidosis with a degree of aortic stenosis in an old patient without amyloidosis proven cardiac involvement)**

***Response:*** *Thank you for your comment. We understand the concern raised, but this is an intrinsic limitation of the available information database used for this study. As mentioned before, we tried to mitigate this limitation according to the recommendations of the expert panel feedback. The deeper evaluation your comment proposed would only be possible using a traditional medical chart retrospective assessment, which would be a different study and objective from ours.*

*In the case of ATTR-CM reference cases, we used the ICD-10 codes, E85.0, E85.1, and E85.2 for hATTR-CM, and E85, E85.3, E85.4, E85.8, and E85.9 combined with cardiac involvement ICD-10 codes. In addition, the same ICD-10 codes were previously published articles, such as Brown et al, 2021 and Jang et al, 2022.*

**In conclusion, I appreciate the effort but I have to express my concern on most of the definitions you provided and consequently to all the analysis derived from them.**

***Response:*** *We appreciate all the concerns and questions raised in the review. We considered all of them and included more descriptions of the model construction, datasets, and population included and tested in the methodology. Also, we added more information in the limitations section regarding the absence of lab exam information.*

**You excluded patients with “blood cancer” to avoid overlap with AL however, all patients with a positive serum or urinary immunofixation rise the suspect of possible AL (independently from the presence of a blood cancer) and only biopsy proven ATTR should be included, I don’t think this has been done in your study, rising additional biases.**

***Response****: We appreciate all the concerns and questions raised by the Reviewer. As mentioned, and explained in the other comments, as this is a claim database study, we have some intrinsic limitations regarding lab results availability. Also, this is also aligned with similar studies using ML performed previously, such as Huda et al, 2021, which is added in the references.*

**Is it normal that in the manuscript I repeatedly found “(Supplementary Error! Reference source not found.)”? Maybe there is something wrong in my downloaded manuscript, but it is difficult to understand the refences in the text.**

*Response: This was a mistake of the reference manager. We have adjusted it in the revised new version. We apologize for that.*

**For me it is unclear how you tested the accuracy, sensitivity and specificity of the ML model, from what I understood you classified wtATTR-CM and hATTR-CM based on ICD-10 with criteria not accepted internationally for the diagnosis of cardiac amyloidosis.**

***Response****: Detailed description included in the methods section and the figure below in the Supplementary material.*

**Supplementary Fig 3. Equations for Accuracy, Sensitivity and Specificity calculation**

TP: true positive; TN: true negative; FP: false positive; FN: false negative

**Reviewer** 2

**In the introduction I suggest to authors to read and refer to this article which explains in general what amyloid fibrils are: Auriemma Citarella, Alessia, et al. "ENTAIL: yEt aNoTher amyloid fIbrils cLassifier." BMC bioinformatics 23.1 (2022): 1-15.**

***Response:*** *Thank you for your suggestion. Reference added.*

**In the introduction I suggest putting a short introduction to cardiovascular disease in general and referring to these papers: De Marco, Fabiola, et al. "Classification of QRS complexes to detect Premature Ventricular Contraction using machine learning techniques." Plos one 17.8 (2022): e0268555. De Marco, Fabiola, Dewar Finlay, and Raymond R. Bond. "Classification of Premature Ventricular Contraction Using Deep Learning." 2020 Computing in Cardiology. IEEE, 2020.**

***Response****: Thank you. We added a short contextualization of cardiovascular and included the suggested references.*

**At the end of the introduction, I suggest inserting a brief description of the objective of the paper, the dataset used, and a very brief description of the results achieved.**

**In the introduction, add structure to the paper to make it easier to read.**

***Response:*** *Thank you for your suggestion. We adjusted the introduction and added more information on the last part of it.*

**Check the references in the method section.**

***Response:*** *There was an error on the reference manager. We adjusted it for this version. We apologize about it.*

**What are the fields of the dataset? How many people are involved in the study? Clarify and include a better description of the dataset.**

***Response:*** *We included a deeper description of the database in the methodology section.*

**A misunderstanding exists between dataset parameters and model input parameters. Are they identical? Are only some selected? Then why?**

***Response:*** *Some of the dataset parameters were used as model inputs, such as age and procedures performed.*

**Why are so many more samples taken from people over 50? Does this occur more frequently in people over 50? Explain this point**.

***Response****: Yes, wtATTR-CM occurs mainly in older populations and this age cutoff was also used in similar studies previously reported. We clarified this point in the methodology section.*

**Insert a diagram or table to explain the structure of the dataset well, explaining what they are for each field and category in order to favor the reader.**

***Response:*** *Thank you for your suggestion. We included a specific reference for this, Ali et al, 2019.*

**What percentage are training, validation and testing chosen?**

***Response:*** *The training, validation and test steps were performed with 60%, 20% and 20% of patients, respectively. This information was added in the methodology and is also available in the supplementary material figure 2.*

**Why wasn't the AUC metric used? please if it is possible to insert it.**

***Response****: The accuracy result generated does not necessarily represent the clinical applicability for the expected result. Despite the universal use in machine learning studies, area under the curve of a receiver operating characteristic curve is not necessarily the best metric to represent clinical applicability and is not easily understandable by many clinicians. Machine learning algorithms will use whatever information are available to achieve the best possible performance in the dataset used. This may include the exploration of unknown confounders that may not be reliable, impairing the algorithm’s ability to generalize to new datasets.*

**The description part of the dataset in the results section should be moved to the dataset subsection.**

***Response****: We improved the dataset description on the dataset subsection and included results of the methodology applied in the results section*

**It is unclear what descriptions images and tables refer to in the results section. Need to double check and fix. In this way I cannot say that they are clear and adequate**

***Response:*** *Thank you for your comment. We have double checked both figures and tables. Also, as the submission system issues the file with figures in separate, usually this makes it harder to evaluate it in the text.*

**The conclusion needs improvement. In this way, they cannot be accepted. The authors should provide a summary of their work, report a portion of the findings, and explain how their work has improved the domain.**

***Response****: Information added in the conclusion section.*

*“In our study, we were able to characterize this population demographically, clinically (considering their ICD-10 codes and procedures performed), and to identify the HCRU related to ATTR-CM management. The use of ML as a tool to identify potential patient of underdiagnosed diseases can be a hallmark for public health resources allocation and medical education strategies. In addition, our findings may be useful to support the development of health guidelines and policies to improve diagnosis, treatment and to cover unmet medical needs of patients with ATTR-CM in Brazil.”*

**REFERENCES**

- Brown D, Vera-Llonch M, Perez J, et al. USE OF COMMERCIAL CLAIMS DATA TO ESTIMATE TRANSTHYRETIN-AMYLOID CARDIOMYOPATHY PREVALENCE AND INCIDENCE IN THE US. J Am Coll Cardiol. 2021 May, 77 (18_Supplement_1) 884. https://doi.org/10.1016/S0735-1097(21)02243-9
- Huda, A., Castaño, A., Niyogi, A. et al. A machine learning model for identifying patients at risk for wild-type transthyretin amyloid cardiomyopathy. Nat Commun 12, 2725 (2021). <https://doi.org/10.1038/s41467-021-22876-9>
- Jang SC, Nam JH, Lee SA, An D, Kim HL, Kwon SH, Lee EK. Clinical manifestation, economic burden, and mortality in patients with transthyretin cardiac amyloidosis. Orphanet J Rare Dis. 2022 Jul 15;17(1):262. doi: 10.1186/s13023-022-02425-3. PMID: 35840997; PMCID: PMC9287852.
