# Supplementary material for: Assessment of potential transthyretin amyloid cardiomyopathy cases in the Brazilian public health system using a machine learning model
Source: PLoS One. 2024 Feb 15;19(2):e0278738. doi: 10.1371/journal.pone.0278738 (PMC10868784; doi:10.1371/journal.pone.0278738)
Supplement: Attachment — Submitted filename: Response to Reviewers_r2.docx [file pone.0278738.s012.docx]

**Reviewer**

1. **Review punctuation (spaces after brackets).**

***Response:*** *Thank you for your suggestion. We reviewed all the spaces after brackets.*

2. **Review the reference in line 309.**

***Response:*** *Thank you! We reviewed all the references in the article.*

3. **Add a figure to help understand the composition of the dataset visually.**

***Response:*** *Thank you for your suggestion, we included one figure as supplementary figure 2.*
